# A Systematic Review of Parkinson’s Disease Cluster Analysis Research

**DOI:** 10.14336/AD.2021.0519

**Published:** 2021-10-01

**Authors:** Renee M Hendricks, Mohammad T Khasawneh

**Affiliations:** Department of Systems Science and Industrial Engineering, Binghamton University, Binghamton, NY 13902, USA.

**Keywords:** Cluster Analysis, Parkinson’s Disease, Patient Subgroups

## Abstract

One way to understand the Parkinson’s disease (PD) population is to investigate the similarities and differences among patients through cluster analysis, which may lead to defined, patient subgroups for diagnosis, progression tracking and treatment planning. This paper provides a systematic review of PD patient clustering research, evaluating the variables included in clustering, the cluster methods applied, the resulting patient subgroups, and evaluation metrics. A search was conducted from 1999 to 2021 on the PubMed database, using various search terms including: Parkinson’s disease, cluster, and analysis. The majority of studies included a variety of clinical scale scores for clustering, of which many provide a numerical, but ordinal, categorical value. Even though the scale scores are ordinal, these were treated as numerical values with numerical and continuous values being the focus of the clustering, with limited attention to categorical variables, such as gender and family history, which may also provide useful insights into disease diagnosis, progression, and treatment. The results pointed to two to five patient clusters, with similarities among the age of onset and disease duration. The studies lacked the use of existing clustering evaluation metrics which points to a need for a thorough, analysis framework, and consensus on the appropriate variables to include in cluster analysis. Accurate cluster analysis may assist with determining if PD patients’ symptoms can be treated based on a subgroup of features, if personalized care is required, or if a mix of individualized and group-based care is the best approach.

## Background

Parkinson’s disease (PD) was first described in 1817 in a publication by Dr. James Parkinson, a British physician [1]. PD is the second, most common neurodegenerative disorder [2]. It is a chronic and progressive disease. No treatment stops the progression, and its cause(s) are unknown. Symptoms cover multiple, functioning areas, making it the umbrella of disorders.

Clinical scales are utilized to quantify disease progression and severity [2]. A series of clinical scales are utilized to evaluate PD patients, with the Unified Parkinson’s Disease Rating Scale (UPDRS) the most, globally recognized scale [3]. But many of these PD clinical scales are ordinal in type, with their resulting scores also ordinal, not providing a quantifiable, progression nor severity level, even though cluster studies utilized these scale results to define disease progression and severity levels for a multitude of symptoms.

The clinical variability between patients with Parkinson’s disease may point at the existence of subtypes of the disease, and identification of subtypes is important, since a focus on homogeneous groups may enhance the chance of success of research on mechanisms of disease and lead to tailored treatment strategies [4]. In addition, defining subtypes (or clusters) of PD is needed to better understand underlying mechanisms, predict disease course, and eventually design more efficient, personalized management strategies [5].

**Table 1:** Hoehn & Yahr Scale and Modified Version [6].

Hoehn and Yahr scale	Modified Hoehn and Yahr scale
1: Unilateral involvement only usually with minimal or no functional disability	1.0: Unilateral involvement only
	1.5: Unilateral and axial involvement
2: Bilateral or midline involvement without impairment of balance	2.0: Bilateral involvement without impairment of balance
	2.5: Mild bilateral disease with recovery on pull test
3: Bilateral disease: mild to moderate disability with impaired postural reflexes; physically independent	3.0: Mild to moderate bilateral disease; some postural instability; physically independent
4: Severely disabling disease; still able to walk or stand unassisted	4.0: Severe disability; still able to walk or stand unassisted
5: Confinement to bed or wheelchair unless aided	5.0: Wheelchair bound or bedridden unless aided

## Parkinson’s Disease Clinical Scales

One of the earliest scales for PD assessment is the Hoehn & Yahr (HY) Staging Scale, which was developed to be a simple scale to provide an estimate of clinical function, combining disability and impairment [6]. The main focus of the scale is differentiating unilateral versus bilateral symptoms and presence or absence of balance issues [2]. Furthermore, in the early 1990s, 0.5 increments were added, with these scales (in Table 1) currently utilized for clinical trials to determine patient inclusion.

**Figure 1.**
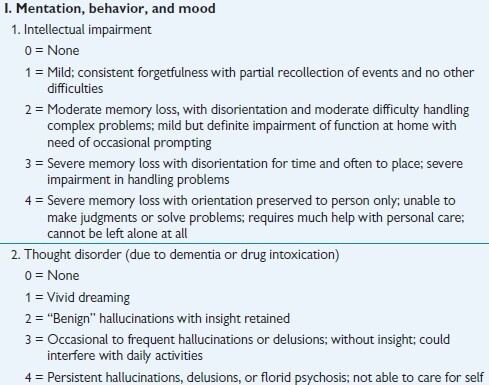
UPDRS Part I, Questions 1 and 2 [2].

The UPDRS was developed in the mid-1980s, to incorporate elements from existing scales in order to provide a comprehensive way to monitor PD disability and impairment [7]. Furthermore, this scale consists of the following four sections: Part I: Mentation, Behavior and Mood; Part II: Activities of Daily Living; Part III: Motor; and Part IV: Complications (of treatments). Parts I and II can be completed by the patient and/or their caregiver. Parts III and IV is rated by a clinician. The scores in each section are added up and then summed together to provide the total score, referred to as the Total-UPDRS. The sum of Part III (motor skills) is also reported and referred to as Motor-UPDRS.

The Modified Hoehn and Yahr scale and the Schwab and England scale are included as supplemental surveys. Schwab and England’s Activities of Daily Living scale is a 10-point scale that rates disability in performing daily activities, with a high score of 100% equating normal function and the lowest score of 0% representing total dependency [2]. For demonstrative purposes, Figure 1 displays UPDRS Part I, Questions 1 and 2.

**Figure 2.**
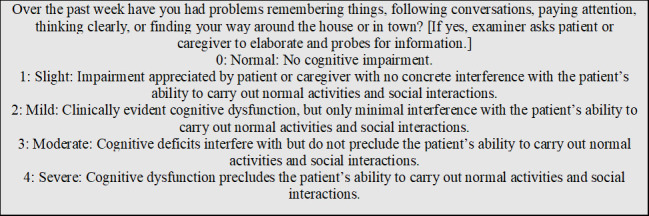
MDS-UPDRS Part I, Question 1.1 [8].

The Movement Disorder Society (MDS) developed and published the revised scale, MDS-UPDRS, in 2008. This scale focuses on symptoms’ impact, not presence as seen in the UPDRS. New areas of assessment were added and include anxious mood, urinary problems, constipation, fatigue, and getting in and out of bed [8]. For demonstrative purposes, Figure 2 displays Question 1.1 from the MDS-UPDRS Part I.

It was stated in [9] that PD assessment scales are subjective, inferential, based on rater-based interview and examination and patient self-assessment, consisting of rating scales and questionnaires that provide estimations of conceptual, non-observable factors (e.g., symptoms), usually scored on an ordinal scale. Successive categories do not represent equal differences of a measured attribute, and the resulting data is ordinal, nonmetric, and categorical, with appropriate statistics for this type of data consisting of only average, mode, median and frequency distributions [10]. Observing question excerpts of the UPDRS and MDS-UPDRS in Figures 1 and 2, one can see these scales, along with the HY Staging Scale (Table 1), are of an ordinal type as the choices are successive, categorical labels. The labels do not represent equal differences, and at times the choice descriptions appear equal with choice 3 and 4 referring to severe in the UPDRS (Fig. 1). Consistence choice labeling has been incorporated into the MDS-UPDRS, but it is still an ordinal scale with categorical labeling. In addition, a selection of choice 4 does not mean this choice description is twice as severe as choice 2, in any of the scales.

## Literature Review Methodology

This study follows the PRISMA guidelines to help research develop and organize a systematic literature review [11]. The focus of this review is solely based on PD patients, not developing PD and non-PD patient subgroups, with patient subgroups determined through cluster analysis and the use of variables defined by clinical features.

More specifically, the objectives of this review are to:

1. Identify the variables (disease features, demographics, etc.) included in clustering, the cluster methods applied, and the resultant patient clusters, and
2. Evaluate and emphasize differences and similarities among the studies, and
3. Determine research gaps and future directions.

The exclusion criteria included the following:

**Exclusion Criteria 1**: Published book sections, reports, and theses.

**Exclusion Criteria 2**: Working papers and articles under review by December 2020, and

**Exclusion Criteria 3**: Articles without access to the whole paper.

**Figure 3.**
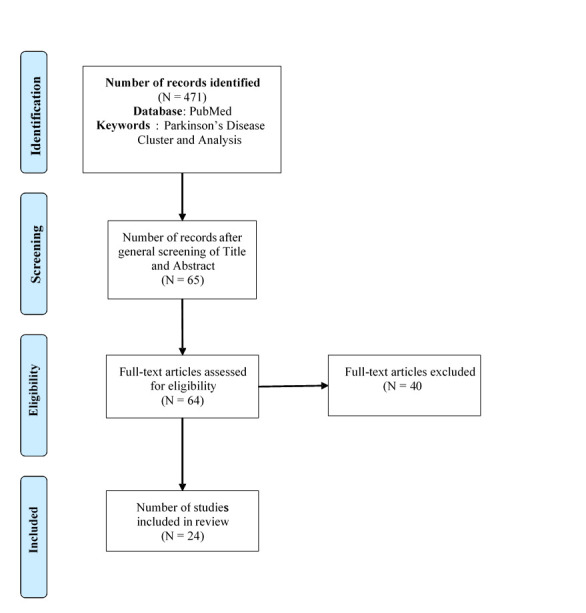
Literature Search Strategy Process.

This research queried the PubMed database, using various search terms including: Parkinson’s disease, cluster, and analysis, between the years of 1990 - 2021, which identified 471 papers. Based on the criteria, 406 papers were excluded after screening titles and abstracts of the 471 papers, as many were focused on other neurological conditions, not focused on clustering, or focused on providing an overview of PD. From the remaining 65 articles, 40 were excluded because of the focus of one symptom (or domain) for clustering, such as gait or cognition, and for one instance, the entire article was not accessible, providing a total of 24 for this review. Studies that applied cluster analysis methods through the use of an algorithm or manual grouping of patient information were included. Figure 3 depicts the literature review search strategy process following the PRISMA guidelines.

**Figure 4.**
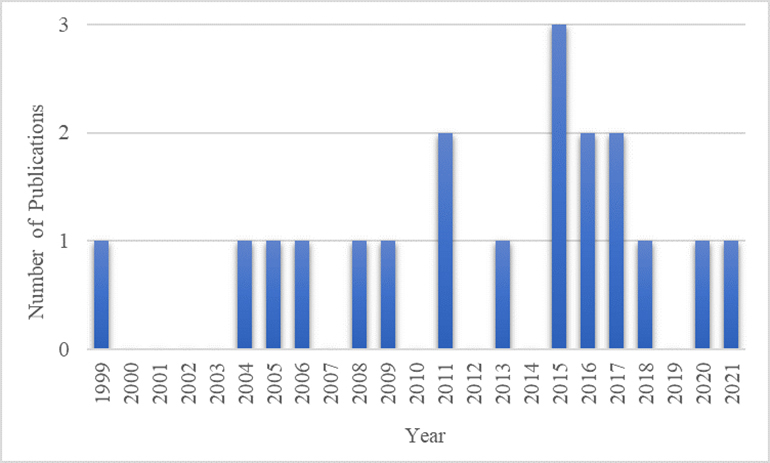
Number of Parkinson’s Disease Cluster Analysis Publications Per Year.

The number of publications per year are displayed in Figure 4. In 2015, the number of publications peaked, with 3 publications that year, with 2 publications in the years 2011, 2016, and 2017.

The following sections explores in detail these discovered review and research articles, the variables included in clustering, the data pre-processing and reduction methods applied prior to clustering, the clustering techniques and their limitations, the similarities found among the resulting clusters, the limitations in the evaluation of the resulting clusters, and concluding with the path forward for future cluster analysis of PD patients.

## Review Articles

Of the twenty-four discovered articles, four consisted of review articles, listed in Table 2. These reviews validated the literature search results, provided a baseline for article summaries and an additional article not found through the PubMed search. Three of the articles provided summaries and comparisons of clustering results, with the 4th article evaluating the reproducibility of past cluster results. In this article, eight cluster analysis results were evaluated by a panel of experts, using a modified Delphi consensus process, but after two iterations, no study was reproducible, providing the conclusion that data-driven PD subtype classification systems lack reproducibility [12]. In addition, these authors raised concerns about the utility of data-driven PD subtypes and call for the establishment of standards for the validation and use of these subtype classification systems.

**Table 2:** PD Cluster Review Studies.

Reference	Review Period	Number of Reviewed Studies	Studies Beyond Previous Review Article	Focus
van Rooden et al. (2010)	1999-2010	7	-	Patient Clusters
Marras and Lang (2013)	1999-2012	9	2	Patient Clusters
Mestre et al. (2018)	1999-2015	10	1	Validation of Clusters
Qian and Huang (2019)	2011-2017	6	3	Patient Clusters

The search strategy of [4] was repeated in [13] and expanded to include studies published up to May 2012, providing 9 total studies, including the 7 studies evaluated in [4], with the addition of two studies: [14-15]. The focus of [12] was to identify all published studies of data-driven PD subtype classification systems and attempt to reproduce the cluster analyses of these studies in their patient cohort. As part of the review process, 10 studies were identified, including the nine from [13], with one additional paper, [5]. Then, [16] conducted a review to analyze existing subtypes of Parkinson’s disease and discovered six PD cluster studies with the addition of three studies [17, 18, 19], not previously noted in the earlier reviews, providing a total of 13 studies among the review papers. These thirteen studies were discovered in this review process, with the addition of seven studies, providing a total of twenty studies explored in this paper.

## PD Patient Cohorts

Six studies included data from PD patient cohorts in the analysis, including:

• PROfiling PARKinson’s (PROPARK) and Estudio Longitudinal de pacientes con Enfermedad de Parkinson cohorts [14],
• Parkinson’s Progression Markers Initiative (PPMI) [17, 18],
• Oxford Parkinson’s Disease Centre Discovery and Tracking Parkinson’s [20] and [21]),
• Non-Motor Symptoms Scale (NMSS) and Non-Motor International Longitudinal Study (NILS) cohorts [19].

The PROfiling PARKinson’s (PROPARK) is a Dutch cohort of 344 PD patients, whereas the Estudio Longitudinal de pacientes con Enfermedad de Parkinson cohort (ELEP) consisted of a Spanish cohort of 257 patients [14]. Tracking Parkinson’s is a cohort of PD patients recruited in the UK between Feb. 2012 and May 2014 [20]. The Oxford Parkinson’s Disease Centre Discovery cohort are PD patients recruited from 11 hospitals in the Thames Valley region between Sept. 2010 and Jan. 2016, and these cohorts were predominantly white [21].

The characteristics of the patients included in cluster analysis are summarized in Table 3. Nine studies (noted with an asterisk) included longitudinal patient data. The patients’ mean or median age and disease duration, along with the number or percentage of male and female patients is included. All datasets contained more male patients than female patients, with an age range of 57.47-73.2 years among the studies, and a disease duration range of 6.5 months-11 years. These limited ranges may affect defining differences among the clusters.

## Patient Variables Included in Clustering

Nineteen of the twenty studies included the PD clinical scale scores in the clustering. Three studies included the HY, eleven studies included the UPDRS, and five included the MDS-UPDRS scores for motor and non-motor symptom severity. In addition to the PD clinical scales, a total of forty-eight scales, questionnaires, and exams, were discovered among the studies, of which the resulting score(s) were included in the cluster analysis. These assessment tools are listed in alphabetical order in Table 4.

After review and collection of the assessment tool names and abbreviations, an internet search was conducted to verify the names and abbreviations to provide a concise and accurate listing in Table 4. For example, the Stroop word-colour test was listed in a study, but after a search, only the Stroop Color/Colour Word Test was found, and this test was renamed as such. A Leeds Anxiety and Depression Scale (LADS) was listed in one of the studies, but from the internet search, the Leeds scales for the self-assessment of anxiety and depression was discovered and hence, this is listed in its place. In addition, a flamingo test was listed in one of the studies and after a search, a flamingo balance test was discovered, so the word balance is included.

**Table 3:** Patient Characteristics in Cluster Analysis Research (*=longitudinal data).

Reference (in year order)	# of Patients [Male (M), Female (F)]	Mean Age of Patients (Years)	Mean Disease Duration (Years)
Graham and Sagar (1999)	176 [93 M, 83 F]	63.2	7.5
Gasparoli et al. (2002)	103* [69 M, 34 F]	-	-
Dujardin et al. (2004)	44*	Median: 66	Median: 1
Lewis et al. (2005)	120 [77 M, 43 F]	64.4	7.8
Post et al. (2008)	133* [54 M, 46 F]	66.7	20 months
Reijnders et al. (2009)	346 (Cohort 1: 224, Cohort 2: 122)	73.2 (1), 65.3 (2)	9.0 (1), 6.7 (2)
van Rooden et al. (2011)	226 M, 118 F (1) * 184 M, 92 F (2) * 193 M, 164 F (3) *	60.8 (1) 61.5 (2) 66.2 (3)	9.9 (1) 11.0 (2) 7.7 (3)
Liu et al. (2011)	138 [80 M, 58 F]	57.47	3
Erro et al. (2013)	100 [59 M, 41 F]	59.7	13.4 months
Lawton et al. (2015)	769* [508 M, 261 F]	64.77	2.92
Ma et al. (2015)	1,510	66.7	63.9 months (5.3 years)
Fereshtehnejad et al. (2015)	113* [73 M, 40 F]	66.7	5.7
van Balkom et al. (2016)	226 [65% M, 35% F]	63.4	3
Fereshtehnejad et al. (2017)	421* [276 M, 145 F]	61.1	6.5 months
Mu et al. (2017)	904* [62.17% M]	64.38	8.0
Lawton et al. (2018)	1,601* [ 1,047 M, 554 F] 944 [610 M, 334 F]	65.9 (1) 65.9 (2)	1.3 (1) 1.2 (2)
Belvisi et al. (2021)	100 [65 M, 35 F]	63.4	1.3

The SCOPAs cover a variety of topics including cognition, motor function, and nighttime sleep problems and excessive daytime sleepiness. A limit set of scales focused on patient motor features including finger tapping ability, the ability to get up, speech, and motor function. The majority of tools are designed for assessing non-motor symptoms, with six focused on depression, six focused on sleep, four focused on anxiety, and two focused on dementia. Does the variety of scales point to a lack in framework in assessing non-motor symptoms? Which scale(s) are to be utilized and in what order? The UPDRS and MDS-UPDRS contains assessment questions for non-motor symptoms, but are supplemental tools required?

One study [22], included the interference cost index of the Stroop Colour and Word Test (SCWT), the number of different words named in alternating and semantic word fluency tests, the number of words correctly free recalled, free and cued recalled and delayed free recalled in the G&B test. [23] The SCWT inference measure for color-word task tile corrected for color-only time was also included in [23]. In addition, [23] included the Trail Making Task (TMT) task B time corrected for task A time, the backward digit span subtest of the Wechsler Adult Intelligence Scale (WAIS)-III, both which evaluate short-term memory, and a 15-min delayed recall of a 15-word list learning task (abbreviated as 15WT) Dutch version of the Rey Auditory Verbal Learning Test, which evaluates long-term memory.

A genetic risk score, visuospatial, speed/attention, memory, and executive function cognitive scores were included in [18]. In addition, levodopa dose equivalents, responses and complications were included as variables in limited studies. Three studies included orthostatic blood pressure measurements or drops.

Studies [24] and [15] utilized a motor phenotype score which consisted of obtained by dividing the patient’s tremor score by their non-tremor score from the UPDRS. The tremor score was the sum UPDRS items 16 and 20 - 26, divided by 8, which represented the degree of tremor reported in the activities of daily living section of the UPDRS, along with tremor at rest and in action, determined on physical examination. The non-tremor score was derived from the sum of items 5, 7, 12-15, 18, 19, and 27-44 on the UPDRS divided by 26.

**Table 4:** Assessment Tool Results Included in PD Patient Clustering.

Alternate Finger Tapping Test (a-FTT)	Movement Disorder Society-Unified Parkinson’s Disease Rating Scale (MDS-UPDRS)
Beck Anxiety Inventory (BAI)	National Adult Reading (NAR) Test
Beck Depression Inventory (BDI) *	Non-Motor Symptoms Questionnaire (NMSQuest)
Benton Judgement of Life Orientation (JLO)	Non-Motor Symptoms Scale (NMSS)
Big Five Inventory (BFI)	Pattern recognition memory (PRM)
Blessed Dementia Scale	Pittsburgh Sleep Quality Index (PSQI)
Cambridge Neuropsychological Test Automated Battery (CANTAB)	Purdue Pegboard Test
Clinical Impression of Severity Index for PD (CISI-PD)	Questionnaire for Impulsive-Compulsive Disorders in PD (QUIP)
Epworth Sleepiness Scale (ESS)	REM Sleep Behavior Disorder Screening Questionnaire (RBDSQ)
Fatigue Severity Scale (FSS)	Rey Auditory Verbal Learning Test
Flamingo Balance Test	Scales for Outcomes in Parkinson’s Disease - Autonomic Dysfunction (SCOPA-AUT)
Freezing during on, speech, and swallowing (FOSS)	Scales for Outcomes in Parkinson’s Disease - COGnition (SCOPA-COG)
Frontal Assessment Battery (FAB)	Scales for Outcomes in Parkinson’s Disease - Motor Function (SCOPA-Motor)
Geriatric Depression Scale (GDS)	Scales for Outcomes in Parkinson’s Disease - Psychiatric (SCOPA-PC)
Grober and Buschke (G&B) test	Scales for Outcomes in Parkinson’s Disease - Sleep (SCOPA-Sleep)
Hamilton Depression Rating (HAM-D) Scale (HDRS) *	Sniffin’ 16 odour identification scores
Honolulu Asia Aging Study Constipation Questionnaire	State-Trait Anxiety Inventory for Adults (STAIT)
Hopkins Verbal Learning Test (HVLT)	Stroop Colour and Word Test (SCWT)
Hospital Anxiety and Depression Scale (HADS)	Symbol Digit Modalities Test (SDMT)
Hoehn & Yahr (HY) Staging Scale	Timed Up and Go Test
Leeds scales for the self-assessment of anxiety and depression	Tower of London (TOL) test
Mattis Dementia Rating Scale (MDRS)	Trail Making Test (TMT)
Mild Cognitive Impairment (MCI)	Unified Parkinson’s Disease Rating Scale (UPDRS)
Mini-Mental Status Exam (MMSE) *	University of Pennsylvania Smell Identification Test (UPSIT)
Montgomery - Asberg Depression Rating Scale (MADRS)	Wechsler Adult Intelligence Scale (WAIS)-III
Montreal Cognition Assessment (MoCA)	

The presence of the following motor and non-motor symptoms was included in cluster analysis: bradykinesia, constipation, dementia, motor fluctuations dyskinesias, Postural-Instability-Gait-Difficulty (PIGD), rigidity, and tremor. Disease progression was calculated in five studies as dividing the UPDRS clinical score by disease duration: [15, 24-27]. In addition, [28] defined progression as the HY score per year and [29] defined progression solely with the HY score.

Age of PD onset values were included in half the studies (ten) and disease duration values were included in six studies. Categorical variables of gender, family history, and dominant symptom side were reviewed as part of post-analysis of the clusters in a limited number of studies. Only one published study, [30], included gender in the clustering.

The most recently published study, [31], did not utilize clinical scale scores, but measurements and analysis incorporating transcranial magnetic stimulation (TMS) for primary motor cortex and plasticity measurement, kinematic analysis of the fast index finger abduction for motor performance, and somatosensory temporal discrimination threshold (STDT) measurements at rest and during movement, for sensory function. But, utilizing only numerical values may be based on the clustering method utilized, and this will be further explored in the cluster methods section, as existing methods require a distance measurement to determine patient assignments to clusters.

## Pre-Processing and Reduction of Variables

Variables were normalized, standardized, or converted to z-scores, prior to clustering, in ten of the studies. Normalization was conducted in [32]. Clinical scores based on a method referred to as normative were calculated in [18], using % normal age/sex adjusted University of Pennsylvania Smell Identification Test (UPSIT) score, rather than the actual UPSIT score. In addition, these values were then transformed to z-scores. In addition, standardization of variables prior to clustering was conducted by [15, 22, 24, 25, 29].

In addition, variables were transformed to z-scores in [5, 14], and [23]. Prior to the Z-transformations in [14], the assumption was that disease feature severities increase with longer disease durations, and hence, each clinical variable was adjusted for disease duration by obtaining its residual value from a linear regression with the clinical feature as the dependent variable and disease duration as the independent variable. In study [30], data was transformed data such that for each non-binary variable, a direction was determined in that higher values were associated with greater disease severity, defining its direction as = +1, otherwise direction = -1.

Data reduction was applied in four of the studies. The number of variables were reduced prior to clustering through principal component analysis (PCA) in [22]. In addition, studies [20, 21] utilized factor analysis for variable reduction with factors scores and variables not loading into a factor included in cluster analysis. Composite indicators for redundant variables were created for data reduction [18]. For patients with incomplete data, [26, 27, 30] excluded these data points, whereas [20, 23] substituted values (referred to as imputation) to include these data points. Three studies, [14, 18, 21], applied both exclusion and inclusion methods. In [14], if 25% or more of the items of a scale was missing, this patient was excluded from analyses, and for a particular patient with less than 25% of the items of a scale missing, missing data were imputed by the mean value of the non-missing items of that scale of that patient. In addition, [18] imputed missing data using the mean score if 80% or more questions were answered, and in [21], any individual with >20% missing values was excluded, and missing values were imputed by using mean values for the entire cohort. The remaining studies did not note how missing or incomplete data was addressed.

## Determination of Optimal Number of Clusters and Drawbacks

Eight studies determined the optimal number of clusters, providing conflicting results and concern on which to select. [32] determined the number of clusters when the cluster method converged to a 0.01 criterion in nine iterations, resulting in five distinct clusters. The Pseudo F-statistic, Cubic Clustering Criterion, and Squared correlation were utilized in [22]. The Calinski-Harabasz pseudo-F value was utilized to determine the optimum solution to be K = 4, from a selection of K = 3 - 6 in [27]. The authors in [20] arrived at differing conclusions on the optimum number of clusters with the Calinski-Harabasz pseudo-F index pointing to a two-cluster solution, and the Duda-Hart pseudo-T-squared favoring a five cluster solution, when considering models between 2 to 5 clusters.

Optimal K was based on the Gap Statistic and the 1-standard error method in [19]. Most fitting solution (number of clusters and included variables) was selected based on the Bayesian information criterion, in [5]. In study, [23], the number of clusters were determined by a three-phased approach of 1) the ‘best cut’ dendrogram output, 2) the ‘elbow’ in the scree plot and 3) the ecological value of the cluster solution. Of 24 solutions, ten suggested two clusters, and seven suggested three clusters from the results from Hartigan’s rule, in [18].

## Clustering Techniques and Limitations

The two, most common types of clustering techniques are hierarchical (non-partitioning) and partitioning [10]. In partitioning cluster analysis, data is divided into non-overlapping subsets where each data instance is assigned to exactly one subset [33]. However, a drawback is that the user typically specifies the number of clusters as an input parameter [34]. Hierarchical methods do not cluster data directly like partitioning methods, but use grouping or division to gradually assemble or disassemble the data points into clusters [35].

Two common types of hierarchical clustering are agglomerative and divisive. In agglomerative, hierarchical clustering, all points are individual clusters at the starting point, and, at each step, the closest pair of clusters are merged [36]. This step is repeated until all data points are linked together [33]. Agglomerative clustering is a bottom-up approach [34]. In divisive, hierarchical clustering, the starting point is an all-inclusive cluster, and, at each step, splits occur until only singleton clusters of individual points remain. The deciding factor is which cluster to split at each step and how to do the splitting [36].

**Figure 5.**
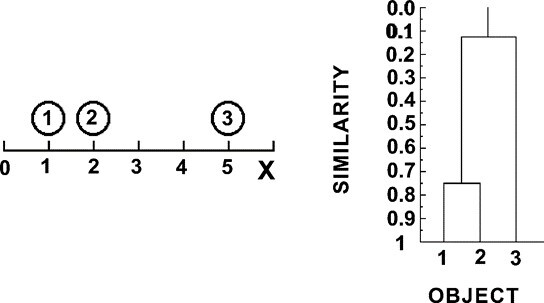
Example Dendrogram [37].

A hierarchical tree (or dendrogram) is constructed to connect all data points at the end [33]. An example dendrogram can be seen in Figure 5 for three objects for one variable [37]. A dendrogram displays both the cluster-sub-cluster relationships and the order in which the clusters were merged, whether agglomerative or divisive [36]. A dendrogram is not utilized to determine the number of clusters but to see the similarity among the data points. A dendrogram is drawn backward, starting from the final cluster with all the objects at a similarity (or distance) of zero. At the similarity where the two clusters merge, the final cluster splits into two-parent clusters and so on. This similarity point occurred at 0.15 (for cluster of 1 and 2 with object 3). The next cluster with points one and two occurs at similarity point 0.75 [37].

**Figure 6.**
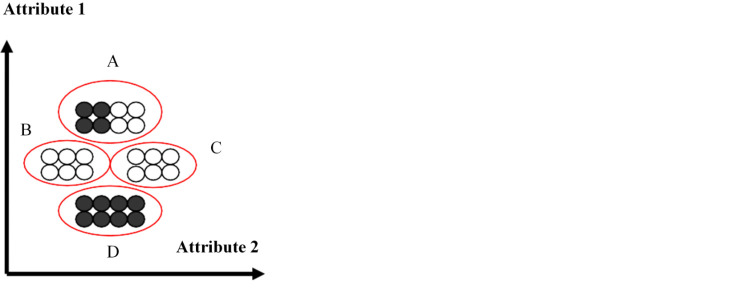
Partitioning Clustering Illustration [39].

In terms of partitioning clustering, K-means clustering is one of the simplest methods, which partitions n observations into k clusters, where k is provided as an input [38]. This method then assigns each observation to clusters based upon the observation’s proximity to the mean of the cluster. The cluster’s mean is then recomputed, and the process begins again [38] Euclidean distance, which is the straight-line distance between any two points, is the proximity calculation in K-means [35]. Figure 6 illustrates partitioning clustering. The black and white examples represent subspecies of Iris plants with four identified clusters, based on two variable attributes [39]. The plants with the highest values for attribute 1 are assigned to cluster A whereas the plants with the lowest value for attribute 1 are assigned cluster D.

The cluster analysis method utilized was not reported in one study, thirteen studies utilized K-means (partitioning) cluster analysis, three utilized hierarchical clustering, one utilized a model-based method (undefined), one incorporated a two-step approach, and one applied a trajectory clustering method. With a series of studies evaluating two to five subtypes, the cluster results ranged from two to five. Two studies concluded with 5 clusters, a majority of studies (nine) reported 4 clusters, five studies reported three clusters, and three studies resulted in two clusters.

The objective of K-means clustering is to minimize distance inside the cluster and maximize the distance between clusters [35]. Because of this, the K-means clustering algorithm applies a distance measurement to cluster the variables, which is not applicable for categorical data types. Hence, only numerical variables were clustered in previous PD studies.

In addition, K-means clustering may not yield the same result with each run, because the resulting clusters depend on initial random assignments [38]. The K-means algorithm does not take into consideration the data distribution and the fact that real objects have no equal importance [35]. This means that data which contain a larger subset, such as a larger male subset, can dominate the cluster outcome because of the larger number of male patient variables. K-means cannot handle clusters of different sizes and densities and has trouble clustering data that contains outliers ([36]). The K-means algorithm may fail to find true clusters in a dataset if there is substantial variability in the data unrelated to differences in clusters. In fact, there is nothing inherent in the K-means algorithm that guarantees that true clusters will be discovered. Instead, the K-means algorithm tends to place sample cluster means where maximal variation occurs in the data [40].

The authors in one study, [30], developed a network-based Trajectory Profile Clustering (TPC) algorithm to group patients based on similar disease trajectory profiles, based on Parkinson’s disease severity variables and the pattern of disease evolution. This method entails constructing a matrix to model the connections between individuals and disease variables and transforming the variables such that variables associated with higher disease severity are replaced with a positive value, and variables associated with lower disease severity are replaced with a negative value. This allows the creation of patient trajectory profiles of individuals who are connected with disease variables for which they have a high enough severity [30].

In addition, [5] referred to a two-step method applied on different combination of variables to improve clustering performance, with the second phase of cluster analysis consisting of utilizing corresponding variables from past studies, including [14, 24, 25, 26, 27, 32, 41], in their cohort.

As noted previously, three studies utilized the hierarchical technique. This method requires few user-specified parameters, and where a user can cut its branches and study the cluster structure at different levels of granularity and detect sub-clusters within clusters, but the resulting clustering may be sensitive to the ordering by which objects are presented [34]. Furthermore, errors in merging clusters cannot be undone and will affect the result, and if large clusters are merged then interesting local cluster structure may be lost.

As noted in [36], hierarchical clustering makes full use of pairwise similarity of all points; however, this approach prevents a local optimization criterion (data-based) from becoming a global optimization criterion (population based). In addition, outliers pose the most serious problems to hierarchical clustering because they distort the cluster centers. Lastly, hierarchical clustering algorithms are expensive in terms of computational and storage requirements.

**Table 5:** Minimum and Maximum Patient Ages Among Clusters.

Reference (in year order)	Minimum Patient Cluster Age (Years)	Maximum Patient Cluster Age (Years)	Age Difference (Years)	Number of Patient Clusters
Gasparoli et al. (2002)	56 (mean)	63.5 (mean)	7.5	2
Lewis et al. (2005)	50	62 (2 Clusters)	12	4
Reijnders et al. (2009)	63.3	75.7	12.4	4
van Rooden et al. (2011)	48.7 (Propark1)	54.9 (mean)	6.2	4
Erro et al. (2013)	54.2	62.3 (at baseline)	8.1	4
Lawton et al. (2015)	61.8	70.4	8.6	5
Ma et al. (2015)	57.3	63.9	6.6	3
Fereshtehnejad et al. (2015)	59.5	63.2	3.7	3
Erro et al. (2016)	60.6	65.4	4.8	3
van Balkom et al. (2016)	55.4	66.3	10.9	4
Fereshtehnejad et al. (2017)	58.3	65.4	7.1	3
Mu et al. (2017)	62.5	71.1	8.6	4

## Limited Age Ranges Among Patient Clusters

Similar patient subtypes were noted in the studies, post-cluster analysis, including old age-at-onset and rapid disease progression and young age-at-onset and slow disease progression, with the addition of minor, intermediate, and severe, motor and non-motor symptom sub-types. Even though older age and young age subtypes were listed, a series of limited age ranges were discovered among the cluster solutions. For example, a two-patient cluster solution contained the mean ages of 56 and 63.5 years [41] and were labeled as early onset and older onset, with a mean age difference of 7.5 years. In addition, one study contained an age onset range of 50-62, a 12-year difference, but this is among 4 clusters, of which two of the patient clusters had an identical age of 62 years of age [24]. The largest difference between the minimum and maximum cluster ages was 12.4 years, among 4 clusters in [26].

In the PROfiling PARKinson’s (PROPARK) cohort study, a mean age onset range of 48.7-54.9 years was discovered providing a 6.2-year difference among 4 clusters [14]. It was also noted in [19] that age differences were not observed in two of the four clusters. The smallest difference in minimum and maximum patient cluster ages was 3.7 years, which was among 3 clusters [5]. Are the limited age ranges a reflection of the patient samples or a feature of Parkinson’s disease, as the onset tends to be in later adult years? The minimum and maximum age ranges and differences are summarized in Table 5, along with the number of patient clusters in each study.

## Limited Disease Duration Ranges Among Patient Clusters

In addition, narrow disease duration ranges were found among the cluster solutions, post- analysis, as viewed in Table 6. One study, [32] reported five clusters, three clusters with short durations (all at 5.6 years) and two clusters with longer durations (both at 13.4 years). Disease duration did not significantly differ between three of four clusters in [14], and two of four clusters in [19]. In addition, disease duration was one month difference among patient clusters in [27], 6 months in [20], 1 year difference in [5], and a 4-year difference among 4 patients clusters, with 2 of the clusters with identical 2-year disease durations in [23]. Two clusters were discovered in [31] with similar disease durations of 1.2 and 1.4 years, a 0.2-month difference. With the limited disease durations, future studies may require patients with a variety of disease durations for clustering. The minimum and maximum disease durations and differences are summarized in Table 6, along with the number of patient clusters in each study.

The patient datasets contained limited age ranges and disease durations. These similarities may explain why the cluster results contained limited age and disease duration ranges when the sample population has limited ranges. This may point to the need to exclude variables with limited ranges or similarities, in order to be able to distinguish true differences among patient groups.

**Table 6:** Minimum and Maximum Disease Durations Among Patient Clusters.

Reference (in year order)	Minimum Disease Duration (Years)	Maximum Disease Duration (Years)	Difference in Duration (Years)	Number of Patient Clusters
Graham and Sagar (1999)	5.6 (3 clusters)	13.4 (2 clusters)	7.8	5
Erro et al. (2013)	12.9 months	13.7 months	0.8 months	4
Lawton et al. (2015)	2.7	3.3	6 months	5
Fereshtehnejad et al. (2015)	5.3	6.3	1	3
van Balkom et al. (2016)	2	6	4	4
Belvisi et al. (2021)	1.2	1.4	0.2 years (2.4 months)	2

## Categorical Information Among Patient Clusters

Categorical variables of gender, family history, and dominant symptom side were reviewed as part of post-analysis of the clusters in a limited number of studies. One recently published study included gender in the clustering. As noted earlier, [30] applied a trajectory profile clustering algorithm to group together patients based on the similarities of their disease trajectories. For this application, the gender variable of male and female was converted to binary values of zero for male, and 1 for otherwise, prior to clustering. Subtype 1 contained predominately female patients, who were young, and mixed in terms of severe impairment of motor and autonomic function, mental health and sleep, with good cognition, at baseline and over time. Subtype 3 was defined as the severe subtype, with worse impairment in all domains, in particular motor and cognition, and predominantly male and older, average age.

In the remaining studies, gender and other categorical characteristics were only included in post-analysis of the cluster results with different disease symptoms for predominantly female groups. It was noted in [14] that two clusters (2,4) of four contained more female PD patients than male in PROPARK cohort 1, with one additional patient in one cluster and two additional patients in a second cluster. These clusters both contained patients with pronounced motor complications. In addition, [23] noted one cluster, cluster 3 (of 4 clusters) contained a relatively high proportion of female patients.

Conversely, it was discovered in [20] that patients in group 1 (25.4%) showed a milder form of PD, had a lower average age at onset, with a higher proportion of females, and a lower L-Dopa equivalent daily dose (LEDD), whereas group 2 patients had worse non-tremor motor symptoms, a higher average age and a lower proportion of females. But, upon review of the results table, all clusters contained more male than female patients. In addition, it was noted in [21] that the mild motor and non-motor disease cluster 2 had the highest proportion of women and youngest age at diagnosis. Upon review of the results table, all clusters contained more male than female patients.

Gender was not described or analyzed in the cluster results of [5, 17, 18, 19, 32], but upon review of the corresponding cluster results tables, all study clusters contained more male patients than female patients. Gender was not discussed in [27], but upon review of the corresponding cluster results tables, two clusters contained more male than female patients, with the remaining two clusters containing more female patients.

All datasets contained more male patients than female patients. Hence, the cluster method applied, and the larger subset of male patients can create a male dominance in the cluster results. This may point to the need to analyze male and female patients separately in cluster analysis, to determine if disease signatures are different.

## Cluster Result Evaluations and Limitations

The quality of the cluster separation was assessed through factorial discriminant analysis (FDA) on eight variables retained in cluster analysis, with the projection of individuals on the discriminant function plane showed that the clusters were well separated [22]. Three studies tested the reproducibility of the cluster results on another dataset. The cluster results in the first dataset were tested by evaluating the probability of a cluster membership of patients in a second dataset [26].

The reproducibility of the cluster results for the Dutch cohort, PROPARK 1, was evaluated in the second annual assessment of the same cohort, PROPARK 2, and then further evaluated in an independent, Spanish cohort, ELEP [14]. In addition, discriminant analysis was conducted with the clusters assigned as dependent variables and PD features assigned as independent variables to determine the features which best discriminated the clusters of the PROPARK and ELEP cohorts. With discriminant analysis, motor fluctuations, PIGD, and autonomic dysfunction correctly classified 286 (80%) patients in the ELEP cohort, and when applying the same discriminative variables in the PROPARK cohort (depression instead of autonomic dysfunction), 274 (77%) patients were correctly classified [14].

In addition, [21] utilized a discriminant analysis model to fit the Tracking Parkinson’s clusters and used this to predict clusters within the Discovery cohort. The agreement between the K-means clusters in the Discovery cohort and those predicted by the Tracking Parkinson’s discriminant model provided a low, overall agreement of 67.9% [21]. Applying cluster classification results of one dataset to a second dataset may be of interest, but the importance is that the datasets contain the same data types and values, and both datasets require the same number of clusters. With the discriminant results at low percentages, cluster classifications are not accurate.

Of concern, silhouette scores were not reported in the cluster analysis studies. An average silhouette score is commonly utilized, for both determining the optimal number of clusters prior to analysis and for evaluating cluster results. Cluster validity measures tend to define cohesion, separation, or a combination of these, and can be applied to overall cluster results and individual clusters, with one such measure, the silhouette score, which incorporates both cohesion and separation [36]. A high silhouette score points to similarities among the data points within the clusters.

## Summarization of PD Cluster Research

The PD cluster studies are summarized in Table 7. The number of patients in the studies, the variables included in the clustering, the algorithms applied, and resulting patient clusters are displayed.

**Table 7:** Systematic Review of PD Cluster Analysis Research.

Reference (in year order)	# of Patients	Patient Variables Included in Clustering	Reduction, Preprocessing & Clustering Methods	Patient Clusters / Subgroups / Domains
Graham and Sagar (1999)	176	•Disease duration ?Age at onset ?Alternate Finger Tapping Test (A-FTT) ?UPDRS - Motor, ?Duration: time to dyskinesias, falls and fluctuations ?UPDRS daily activities ?Beck depression inventory (BDI) ?National Adult Reading Test (NART) ?UPDRS mentation ?Blessed dementia scale ?Digit ordering ?Letter fluency ?Cambridge Neuropsychological Test Automated Battery (CANTAB)	Preprocessing: Normalized Clustering: K-means	A total of 5 clusters as follows: Mean Duration: 5 years 1.Good motor control, No cognitive impairment 2.Good motor control, Executive cognitive deficits 3.Older onset age, Poor motor control and complications, Mild cognitive impairment Mean Duration: 14 years 4.Poor motor control, No cognitive impairment 5.Poor motor control, Moderately severe cognitive impairment
Gasparoli et al. (2002)	103	•UPDRS - Motor ?Motor fluctuations presence ?Dyskinesia presence	Clustering: Unknown	1.Slow course, Earlier onset age, Lateralization of parkinsonian signs, Rest tremor presence, Gait disturbance absent (61%) 2.Rapid progression, Older age onset, Absence of lateralization of parkinsonian signs, Predominance of bradykinesia-rigidity and gait disturbance (39%)
Dujardin et al. (2004)	44	•UPDRS - Motor, ?Stroop Colour and Word Test (SCWT) ?Alternating word fluency ?Grober and Buschke test (G&B test) ?Mattis Dementia Rating Scale (MDRS) ?Semantic word fluency	Reduction: Principle Component Analysis (PCA) reduction to eight variables, Preprocessing: Standardized Clustering: K-means	1.No cognitive deficit, Less severe motor symptoms (26 patients) 2.Reduced cognitive efficiency, Exacerbated subcorticofrontal syndrome, Severe motor dysfunction (16 patients)
Lewis et al. (2005)	120	•Age at onset ?Progression: Total - UPDRS / disease duration ?Dopaminergic therapy ?Motor phenotype: Tremor / non-tremor UPDRS ?Mini Mental State Examination (MMSE) ?NART ?Pattern recognition memory (PRM) ?Tower of London (TOL) ?BDI	Preprocessing: Standardized Clustering: K-means K = 2 - 5	1.Young onset 2.Tremor dominant 3.Non-tremor dominant, Cognitive impairment, Mild depression 4.Rapid progression, No cognitive impairment
Schrag et al. (2006)	124	•Age at onset ?Current age ?Dementia presence ?Fluctuations presence ?Dyskinesias presence ?HY ?Progression: HY per year	Clustering: K-means K = 2 - 5	1.Young (mean) onset, Higher depression scores, Taking higher levodopa doses 2.Older onset, Rapid progression, Less often dyskinesias and fluctuations
Post et al. (2008)	131	•Age ?Age of disease onset ?Progression: UPDRS - Motor / disease duration ?Levodopa responsive PD symptoms ?Levodopa non-responsive PD symptoms ?MMSE ?Hospital Anxiety and depressive symptoms (HADS)	Preprocessing: Standardized Clustering: K-means K = 2 & 3	1.Young onset with slow progression 2.Intermediate age onset, More anxiety and depression; 3.Oldest onset, More motor impairment, Higher progression rate
Reijnders et al. (2009)	346 (two cohorts, 224, 122)	•Tremor ?Hypokinesia / rigidity ?Postural-Instability-Gait-Difficulty (PIGD) ?L-dopa complications, ?Apathy and Hallucinations (Both from UPDRS) ?Age at onset ?Cognition: MMSE ?Depressive symptoms: Montgomery - Asberg Depression Rating Scale (MADRS) ?Progression: UPDRS - Total / disease duration	Clustering: K-means	1.Rapid progression 2.Young onset with motor complications 3.Non-tremor dominant and psychopathology 4.Tremor dominant
van Rooden et al. (2011)	Dutch cohort PROPARK1344, PROPARK2 276, Spanish cohort (ELEP) 357	•Motor Complications: *Scales for Outcomes in PD (*SCOPA) - Motor ?Cognitive functioning: SCOPA - COG ?Autonomic symptoms: SCOPA - AUT ?Psychiatric symptoms: SCOPA - PC ?Nighttime sleep problems and excessive daytime sleepiness: SCOPA - Sleep ?Depressive symptoms: PROPARK: BDI ELEP: HADS ?Postural-instability-gait-difficulty (PIGD) ?Freezing during on, speech, and swallowing (FOSS) ?Levodopa dose equivalent (LDE)	Preprocessing: Linear regression residual values for disease duration, Transformation to z-scores Clustering: Model based	1.Mild severity all domains, Younger onset age (largest cluster) 2.Severe, frequent motor complications, Moderately severe sleep and depressive symptoms, Youngest age onset, Large portion of women 3.Intermediate severity in nondopaminergic domains, Higher onset age 4.Severely affected most domains, except mild tremor, High onset age
Liu et al. (2011)	138	•Age ?Age at disease onset ?Disease duration ?H&Y ?Tremor, Rigidity, ?Hypokinesia, PIGD, ?L-dopa complications (all from UPDRS) ?Motor phenotype: Tremor / non-tremor UPDRS ?Cognitive function: MMSE ?Depression: HAM-D ?Pittsburg Sleep Quality Index (PSQI) ?Constipation ?Fatigue severity scale (FSS) ?UPDRS - Total / disease duration, ?UPDRS - Motor / disease duration, ?UPDRS Activities of Daily Living (ADL) / disease duration	Preprocessing: Standardized Clustering: K-means K = 3 - 5	1.Non-tremor dominant 2.Rapid disease progression 3.Young onset 4.Tremor dominant (largest cluster)
Erro et al. (2013)	100	•Motor disability ?Tremor ?Bradykinesia ?Axial (all from UPDRS) ?Time of onset ?Progression: UPDRS / duration ?L-Dopa equivalent daily doses (LEDDs) ?MMSE, ?Frontal Assessment Battery (FAB) ?HADS ?Depression subscale (HADS-D) ?Anxiety subscale (HADS-A) ?Non Motor Symptoms Questionnaire (NMSQuest)	Clustering: K-means Gower method K = 3 - 6	1.Benign pure motor 2.Benign mixed motor-non-motor 3.Non-motor dominant 4.Motor dominant
Lawton et al. (2015)	Oxford Parkinson Disease Centre (OPDC) Discovery Cohort 769	•MDS - UPDRS Parts I and III ?Sniffin’ Sticks 16-item odour identification Test ?Big Five Inventory (BFI) extraversion scale ?Epworth Sleepiness Scale (ESS) ?Rapid eye movement sleep behavior disorder questionnaire (RBDSQ) ?Leeds scales for the self-assessment of anxiety and depression ?BDI, ?Questionnaire for Impulsive-Compulsive ?Disorders in Parkinson’s Disease (QUIP) ?Honolulu Asia Aging Study Constipation Questionnaire ?Montreal Cognitive Assessment (MoCA) ?Phonemic and semantic verbal fluency ?Purdue Peg-board Test ?Timed Up and Go Test ?Flamingo Balance Test ?Orthostatic blood pressure measurement	Reduction: Confirmatory factor analysis Clustering: K-means	1.Mild motor and non-motor disease 2.Poor posture, gait, cognition, smell and postural hypotension, 3.Severe tremor 4.Poor psychological well-being, RBD and sleep 5.Severe motor, non-motor and cognitive disease, with poor psychological well-being
Ma et al. (2015)	1,510	•Age of onset ?Disease progression, ?Stage (HY) ?Motor evaluating scores: UPDRS III, tremor, hypokinesia, rigidity, PIGD ?Non-motor evaluating scores: cognition: MMSE depression: HAM-D sleep disorder: PSQI constipation scores	Preprocessing: Standardized Clustering: K-means K = 3 - 5	1.Non-tremor dominant (NTD, n=469, 31.1%); 2.Rapid progression with late onset (RDP-LO, n=67; 4.4%); 3.Benign pure motor characteristics (BPM, n = 778; 51.5%), without non-motor disturbances; 4.Tremor dominant with slow progression (TD-SP, n = 196; 13.0%).
Fereshtehnejad et al. (2015)	113	•UPDRS Parts II, III ?Orthostatic hypotension ?Mild cognitive impairment (MCI) ?RBDQ ?Depression ?Anxiety	Preprocessing: Transformation to z-scores Clustering: Two-step	1.Mainly motor/slow progression 2.Diffuse/malignant 3.Intermediate
Erro et al. (2016)	Parkinson’s Progressive Marker Initiative (PPMI) 398	•Gender ?Age at onset ?MoCA ?Geriatric Depression Scale (GDS) ?State-Trait Anxiety Inventory for Adults (STAIT) ?University of Pennsylvania Smell Identification Test (UPSIT) ?RBDSQ ?SCOPA - AUT ?Tremor, bradykinesia, motor, rigidity, axial, apathy, fatigue, hallucinations, pain presence (all from MDS - UPDRS)	Clustering: K-means Gower method K = 3 - 6	1.Lowest motor and non-motor burden 2.Motor disability 3.Motor disability, apathy and hallucinations
van Balkom et al. (2016)	226	•UPDRS Part III ?MMSE ?SCWT ?Trail Making Task (TMT) ?Wechsler Adult Intelligence Scale (WAIS)-III ?Dutch version of the Rey Auditory Verbal Learning Test ?Beck Anxiety Inventory (BAI)	BAI, UPDRS-III, MMSE transformed to z-scores Clustering: Hierarchical	1.Young-age (59.9 years) Mildly affected, (N ¼ 86) 2.Old-age (72.3 years), Severe motor and non-motor symptoms (N ¼ 15) 3.(age 64.7 years) Mild motor symptoms, Below-average executive functioning and affective; symptoms (N ¼ 46) 4.(age 64.8 years), Severe motor symptoms, affective symptoms and below-average verbal memory (N ¼ 79)
Fereshtehnejad et al. (2017)	PPMI 421	•Age ?Genetic risk ?Orthostatic systolic ?blood pressure drops ?MDS - UPDRS Parts II and III ?Tremor, PIGD (MDS - UPDRS) ?ESS ?GDS ?STAIT ?QUIP ?RBDSQ ?SCOPA-AUT ?UPSIT ?Visuospatial, speed / attention, memory, executive function cognitive	Reduction & Preprocessing: Normative values, Composite indicators for redundant variables, Transformation to z-scores Clustering: Hierarchical	1.Mild motor-predominant (composite motor and all three non-motor scores below the 75th percentile) (223) 2.Diffuse malignant (composite motor score plus either ≥1/3 non-motor score >75th percentile, or all three non-motor scores >75th percentile) (52) 3.Intermediate (146)
Mu et al. (2017)	904 2 Cohorts as shown below: 1. Non-Motor Symptoms Scale (NMSS) 2. Non-Motor International Longitudinal Study (NILS)	•HY ?NMSS ?Clinical Impression of Severity Index for PD (CISI-PD) ?SCOPA - Motor	Clustering: K-means	1.Mildly affected all domains (largest) 2.Severely affected non-motor domains 3.Severely affected motor domains 4.Severely affected all domains, except tremors
Lawton et al. (2018)	Tracking cohort 1,601 Discovery cohort 944	•MDS - UPDRS ?Big Five Inventory ?ESS ?RBDSQ ?HADS ?Questionnaire for Impulsive-Compulsive Disorders in PD ?Honolulu Asia Aging Study Constipation Questionnaire ?MoCA ?Orthostatic blood pressure measurement ?Sniffin’ 16 odour identification scores	Reduction: Factor analysis Clustering: K-means	1.Fast motor progression, Symmetrical motor disease, Poor olfaction, cognition and postural hypotension, Highest age at diagnosis 2.Mild motor and non-motor disease, Intermediate motor progression, Youngest onset, Large portion of women 3.Severe motor disease, Poor psychological well-being, Poor sleep, Intermediate motor progression 4.Slow motor progression, Tremor-dominant, Unilateral disease
Krishnagopal et al. (2020)	PPMI 194	•Gender ?Age ?MDS - UPDRS ?Benton Judgement of Life Orientation (JLO) ?Symbol Digit Modalities Test (SDMT) ?MoCA ?Hopkins Verbal Learning Test (HVLT) ?Letter number sequencing ?Semantic Fluency Test ?Schwab and England (S&E) ?ADL ?RBDQ ?ESS ?SCOPA - AUT ?GDS ?STAIT	Preprocessing: Transformation Clustering: Trajectory Profile Clustering (TPC)	1.Mixed subtype, Severe impairment of motor and autonomic function, mental health and sleep, good cognition, at baseline and over time, Young and predominantly female 2.Mild subtype, Milder impairment in all domains (motor, cognitive, autonomic and mental) at baseline and over time 3.Severe subtype, Worse impairment in all domains, Older than average, Predominantly male
Belvisi et al. (2021)	100	•Primary motor cortex (M1): Transcranial Magnetic Stimulation (TMS) ?Motor: fast index finger abduction ?Sensory: Somatosensory Temporal Discrimination Threshold (STDT)	Clustering: Agglomerative Hierarchical	1.Mild motor predominant (younger, milder motor, nonmotor symptom severity) n = 76 2.Diffuse malignant (more severe motor, nonmotor manifestations) n = 24

## Discussion and Conclusions

PD patient clustering is a method applied to understand the similarities and differences among patients, which may lead to developments in diagnosis of future patients via pre-defined subtypes and tracking of symptom(s) progression and treatment(s) per patient groups. Further monitoring of patients in clusters may provide discoveries in the movement of patients among clusters and lead to better treatment of their disease pathways. Cluster results in the studies reviewed in this article pointed to two, three, four and five possible, PD patient groups, with the use of different datasets and K values. The selected K value(s) was based on a range of interest, or past studies, not specified, and not specific to the dataset under review. This selection is important as an inaccurately chosen value will provide incorrect patient cluster assignments.

Methods for determining the optimal number of clusters prior to clustering were not cited for many, meaning this step may have been ignored and may explain why some studies applied different number of clusters to the same dataset. The majority of these studies utilized K-means clustering, a commonly applied technique, but it contains a series of limitations, including its application to numerical data, not categorical data. Hence, only numerical variables were clustered in previous PD studies, and hence, categorical variables of family history, or dominant body side affected by the disease were not included in clustering, but utilized for post-analysis of the clusters, for a limited set of studies. This leads to the need for a clustering method to handle PD datasets with mixed variables, as categorical variables may provide insights to PD subtypes. In addition, even though the numerical values of age of onset and disease duration were included, the cluster results pointed to limited ranges for these variables, pointing to the question, if these should be included in clustering or not, if they are similar in value?

In addition, all published studies utilized subjective, nonlinear data from clinical surveys for a multitude of variables for clustering, leading to the need for accurate data for analysis. These clinical scales do not define motor or non-motor symptoms presence and severity, but provide an ordinal scale result. In addition, a set of these studies defined and calculated disease progression by dividing the scale scores by the time since diagnosis. Cluster analysis needs to be conducted without ordinal, subjective scales scores, but with accurate patient demographics, disease symptoms, and treatment outcomes. The Movement Disorder Society Non-Motor Rating Scale (MDS-NMS) was published in 2019, which incorporates a new approach to defining non-motor symptom severity, by calculating a total score which consists of symptom severity multiplied by its frequency [42]. Even though the levels for both frequency and severity are of a successive, categorical, ordinal types, these two values together provide more information and may assist practitioners and patients with understanding their disease progression, and changes in symptoms. For example, knowing that a person selected a severe symptom level provides a starting point, but knowing that a person selected a severe level with a high frequency, provides more information and a way for better separation of severe symptom patients into subtypes. Doing so is considered a manual way of clustering the patients.

Furthermore, silhouette scores were not reported, as average silhouette scores are commonly utilized for both determining the optimal number of clusters prior to analysis and for evaluating the cluster results. A high silhouette score points to similarities among the data points within the clusters. Limited studies attempted to replicate cluster classification results of one dataset to a second dataset, but the importance is that the datasets have to contain the same data types and values, and both datasets require the same number of clusters. It was unknown if this was indeed the case. Future studies with a rigorous design, standardized with respect to the included variables, data processing and clustering analysis technique, may advance the knowledge of PD subtypes [4]. The utility of data-driven PD subtypes calls for the establishment of standards for the validation and use of these subtype classification systems [12].

**Table 8:** Gaps in PD Patient Cluster Research and Proposed Future Research.

Gaps in PD Patient Cluster Research	Proposed Future Research
Clustering with ordinal, nonlinear, subjective, clinical scores	Clustering with accurate, measurable data
Clustering with numerical variables only; No categorical variables included in cluster analysis	Ability to cluster a variety of data types (categorical, numerical and mixed)
Methods require K values chosen arbitrarily or based on previous studies results	A simple method for practitioner use that does not require predetermined K value nor prior knowledge of dataset
No application of existing cluster evaluation methods; Cluster result evaluated by applying cluster labels to a second dataset	Analyze cluster results with existing metrics (i.e., silhouette scores and plots)
Cluster methods do not take into account data distribution or change in data order	Eliminate subset dominance and data order on cluster results
Converge to a local minimum	Converge to a global minimum
Methods may not find true clusters with substantial data variability	Ability to find true and accurate clusters
Methods do not provide explanations as to how the cluster results came about, which also makes the results difficult to interpret	An explainable and interpretable method and results

The gaps discovered in the PD patient cluster studies, including utilization of K-means clustering and numerical variables, and the limitations of the clustering methods including the randomized initiation and predefined input by the user, highlighted earlier from a series of references [4, 10, 33, 34, 35, 36, Tan, 2018] are summarized in Table 8. Based on a review of these gaps, future recommendations for PD patient clustering are proposed and summarized in Table 8. These improvements include the need for a simple, interpretable, and explainable clustering method that does not require prior knowledge or input of the dataset by the end user. In addition, patient clustering methods need to utilize accurate data, handle a variety of data types, or provide ways to transform the different variables, easily for quick analysis, as patient datasets contain a variety of information, and not be affected by a larger subset in the data, such as a larger male patient subset, as their disease features should not overshadow nor dismiss the female patient subset information.
